# CaLRS: A Critical-Aware Shared LLC Request Scheduling Algorithm on GPGPU

**DOI:** 10.1155/2015/848416

**Published:** 2015-02-02

**Authors:** Jianliang Ma, Jinglei Meng, Tianzhou Chen, Minghui Wu

**Affiliations:** ^1^College of Computer Science, Zhejiang University, Zheda Road No. 38, Hangzhou 310013, China; ^2^Zhejiang University City College, Huzhou Road No. 51, Hangzhou 310015, China

## Abstract

Ultra high thread-level parallelism in modern GPUs usually introduces numerous memory requests simultaneously. So there are always plenty of memory requests waiting at each bank of the shared LLC (L2 in this paper) and global memory. For global memory, various schedulers have already been developed to adjust the request sequence. But we find few work has ever focused on the service sequence on the shared LLC. We measured that a big number of GPU applications always queue at LLC bank for services, which provide opportunity to optimize the service order on LLC. Through adjusting the GPU memory request service order, we can improve the schedulability of SM. So we proposed a critical-aware shared LLC request scheduling algorithm (CaLRS) in this paper. The priority representative of memory request is critical for CaLRS. We use the number of memory requests that originate from the same warp but have not been serviced when they arrive at the shared LLC bank to represent the criticality of each warp. Experiments show that the proposed scheme can boost the SM schedulability effectively by promoting the scheduling priority of the memory requests with high criticality and improves the performance of GPU indirectly.

## 1. Introduction

Modern Graphics Processing Units (GPUs) are expected to play a more critical role in general purpose computing tasks ranging from supercomputing machines to hand-held devices. Compared with CPUs, these GPUs have numerous programmable computational cores and they support thousands of active threads running in warp granularity simultaneously. When a warp executes a memory instruction, the threads in the warp will generate data demand. After coalescing, the generated memory requests are sent to the memory subsystem. The warp will be blocked until all data have been met. By switching to other ready warps, GPUs may execute other memory instructions and generate more memory requests. So the memory subsystem is usually flooded with numerous GPU memory requests at any time.

We found that the GPU memory requests have huge diversities in various aspects, including but not limited to the following. (1) Diversity in valid data: some requests may only provide valid data for one thread whereas other requests may service for many threads in the warp. (2) Diversity in the number of ready warps: since the state of GPU pipeline varies from time to time, the number of ready warps when the memory requests are generated will differ from each other. The memory requests with higher ready warps have stronger latency tolerance than others. So, lots of studies use this information to design GPU's memory scheduling algorithms. (3) Diversity in address distribution: the addresses in the memory requests between different GPU cores may be shared or complementary. (4) Diversity in the amount of memory requests in each warp: based on the number of active threads and address coalescing, different warps may produce different amount of memory requests.

For any warp, it cannot resume to execute unless all of the memory requests it generated have been served. In this paper, we aim at exploiting this difference to improve GPU performance. We observed that the SM schedulability can be improved by adjusting the service sequence of memory requests. So we defined a criticality to classify all the GPU memory requests and proposed a memory request scheduling algorithm based on it. We also showed why we design CaLRS on the shared LLC other than global memory or private cache. Overall, this paper makes the following contributions over previous work.

- We discussed the memory scheduling problem on the shared LLC structure in GPU architecture in this paper. In our knowledge, there are only two papers [1, 2] discussed this issue for CMP architectures. The consideration between our work and them are different.
- We tested the memory request queuing situation for the shared LLC banks with various GPU applications and the result shows that there are plenty of memory requests queuing on LLC banks. But current FIFO scheduling policy fails to distinguish the different degrees of importance for the memory requests.
- We showed the relationship between memory request service sequence and SM schedulability. Specifically, the SM schedulability can be improved by adjusting the service sequence of memory requests properly.
- We quantify the importance of a memory request named as criticality by the number of memory requests that originate from the same warp but have not been served when it arrives at the shared LLC bank. Then we proposed a memory request scheduling algorithm based on this criticality information.

## 2. Background

In this section, we describe the baseline GPU architecture and queue structure on shared LLC.

### 2.1. Baseline GPU Architecture

**Figure 1:**
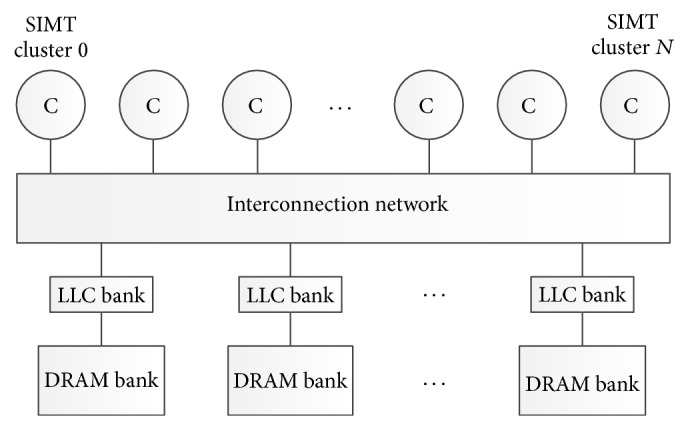
Baseline GPU architecture.

Our baseline GPU shown in Figure 1 consists of multiple cores, named Stream Multiprocessors (SMs). Each SM includes multiple processing units and private data cache. In addition, each SM has another software managed scratchpad memory that is shared by all threads in a thread block. All SMs are connected to a shared LLC (L2 in this paper) via an on-chip interconnection network. The LLC is banked and each bank connects to an off-chip memory channel (memory partition). Each memory channel has a GDDR5 memory partition with a memory controller. We use GPGPU-Sim (v3.2.2) [5] to simulate the baseline GPU (Fermi-like) architecture as depicted in Table 1.

In each SM, the threads pipeline in warps (aka “wavefronts”). A warp consists of up to 32 threads but the active thread number may vary from time to time. Since we study the memory request scheduling algorithm in this paper, we only concern the warps when they execute memory instructions. When a warp executes a memory instruction, we call the warp as memory warp. The active threads in the warp will generate data requirements. If all the threads in the memory warp access addresses within a single cache line range, the data requirements can be coalesced into a single memory request by the Coalescing Unit in each SM. Otherwise the warp has to generate multiple memory requests to access different cache blocks, which is known as GPU memory divergence. But for each cache block, each warp can produce at most one memory request. The memory requests are firstly issued to the private cache in FIFO order. If missed, they are transmitted to the shared LLC. For description simplicity, the following mentioned memory requests denote to the memory requests after coalescing, except any otherwise stipulated.

### 2.2. Shared LLC Queue Structure

The memory requests that have not yet been served in the private cache will be sent to the shared LLC. Port conflicts may happen when lots of memory requests from different SMs arrived at shared LLC simultaneously. Our baseline GPU architecture has 30 SMs in total, but the shared LLC only has 6 banks. So each LLC bank has to serve 5 SMs intuitively. Besides, the number of concurrent threads on each SM can be up to 1536. Even though the memory access rate of each thread may low in some GPU applications, many concurrent threads present in all SMs can still produce numerous memory requests. The small capacity of private cache can only service a small part of memory requests, which leads the shared LLC to intensively competition. In light of the fact that each bank in the LLC serves and schedules memory requests independently, we present the design detail for just one bank.

## 3. Observation and Analysis

### 3.1. The Intensity of the LLC Queue

When more than one memory request arrives at LLC at the same cycle, we say that port competition happens in LLC bank. At these moments, waiting is necessary for some memory requests. We use waiting ratio (WR) to stand for the percentage of waiting, aka, the percentage of moments when more than one memory request arrives at LLC at the same cycle. In this paper, we use a queue to buffer these memory requests. So the length of the queue will be added if the number of new incoming memory requests is greater than the number of issued memory requests. We use average queue length (AQL) to stand for the average value of the LLC queue length when the LLC queue is not empty.

**Figure 2:**
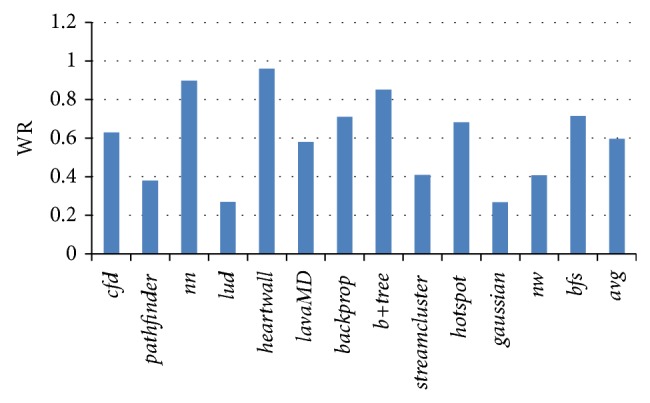
LLC queue waiting ratio.

Figure 2 lists the LLC queue WR for Rodinia [6] benchmarks, in which the last column is the average value. The result shows that much of the benchmarks have high waiting rate, especially for memory intensive benchmarks, such as* nn*,* heartwall*, and* b+tree*. The average waiting ratio of all applications is 0.596, more than a half. The minimum value happened in* gaussian* as 0.267 and the maximum value happened as 0.959 in* heartwall*. Overall, the average possibility for a memory request to wait in the LLC queue is as much as 59.6%. So we can say that the shared LLC is usually busy in GPGPU architecture.

**Figure 3:**
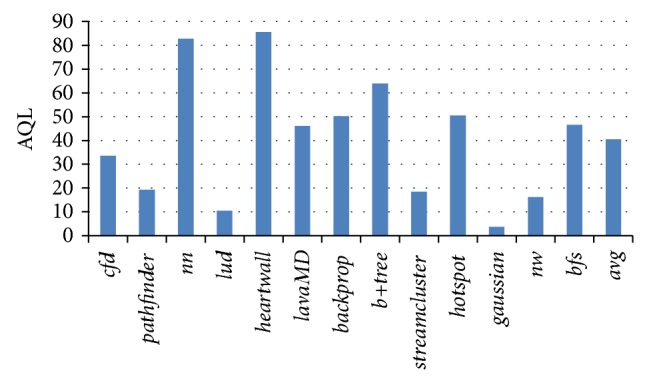
Memory request queue length on shared LLC.

Figure 3 shows the AQL of LLC queue, in which the queue size is set to 100. The average queue length means how many memory requests ahead are waiting in the LLC queue in average. The longer the AQL is, the longer the memory request has to wait according to FIFO sequence. As we can see from the figure, the AQL of each application varies a lot, from 3.66 for* gaussian* to 85.6 for* heartwall*. Besides, the average AQL is 40.57. Based on the test, we can conclude that most of the benchmarks have a lot of request pressure on the shared LLC. The bigger the AQL is, the more intensive the LLC is and the greater the performance potential we can get by optimizing the service sequence of the LLC queue is.

### 3.2. Memory Request Service Sequence Influences the SM Schedulability

**Figure 4:**
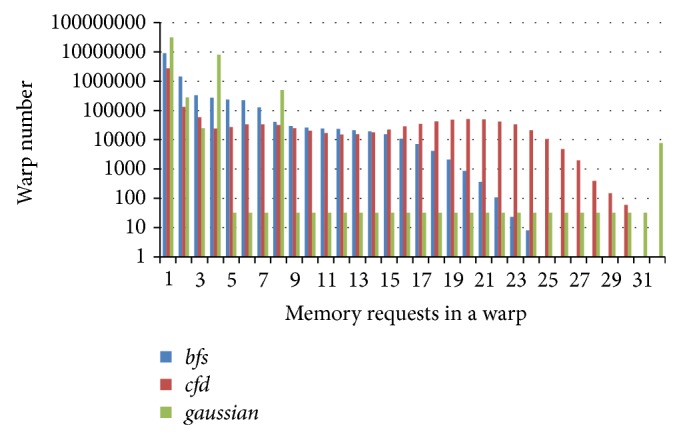
The memory divergence statistic of several benchmarks.

Figure 4 demonstrates the memory divergence statistics of several GPU applications. We can see that a memory warp in* bfs* may produce 1 to 26 memory requests. Although a major part of the memory warps generates only a single memory request after coalescing, the other situations are also ubiquitous. In the worst case, a memory warp has to wait for data result of 26 memory requests in* bfs*. In these situations, the memory subsystem is infeasible to serve so many memory requests in a relative short time under the circumstance of thousands of warps. The situation is even worse in* cfd* and* gaussian* where a warp may generate up to 31 and 32 memory requests, respectively.

We denote SM schedulability as the number of ready warps that can be scheduled at next time. So the stronger the SM schedulability is, the more likely the warp scheduler selects a better warp. For a memory warp, if it has fewer memory requests, it is more likely to obtain all data in shorter time and contribute to SM schedulability. Otherwise, the latency will be high. By scheduling these memory requests in different sequence, the SM schedulability will be influenced. According to this idea, a simple and effective method to improve SM schedulability is to accelerate the data access speed for memory warps that have fewer memory requests. That is to say, the warp with fewer memory requests should have higher priority than others. So, for* bfs* benchmark in Figure 4, the warps with only 1 memory request have the highest priority and the warps with 26 memory requests have the lowest priority.

We have to mention that half completed warp cannot resume to execute. So the interference of the memory request from different warps will worsen both warps. Pervious work [7] proposed an idea that interwarp interference should be avoided. The idea is also suitable for our LLC scheduling algorithm. So we would better gather the memory requests as much as possible by giving them the same scheduling priority.

### 3.3. Design Hierarchy Consideration

As described above, we find that we can improve the SM schedulability by properly adjusting the memory request service sequence. So it is motivated to design an effective memory request scheduling algorithm. In our GPU architecture, there are three places to do this, namely, private cache in each SM, the on-chip shared LLC cache, and off-chip global memory. The private cache is connected with the coalescing logic that merges data requirements from all threads in a warp into minimal number of cache-line-sized memory requests. So adjusting the memory request service sequence in private cache hierarchy equates to adjusting the sequence at the time it is generated. But it is not reasonable. Firstly, the queue is short in this position. As private cache can serve a memory request in every cycle, the memory requests from last memory warp have usually been issued to private cache when the next memory warp arrives. So the contention of memory requests from different memory warp rarely happens. Secondly, the access latency of private cache is similar to that of register file. Then adjusting the memory request service sequence at private cache hierarchy cannot gain much benefit to SM schedulability.

While in the global memory level, after the filtration of LLC structure, the complicated interactions have been diminished, the warp differences are weakened and the physical characters are strengthened. In our GPU architecture, the relationship between LLC bank and memory controller is 1-on-1, so there is no port confliction on memory controller. Besides, the memory queue is formed by the access latency difference between the DRAM and LLC. So memory scheduling algorithms in this level mainly focus on how to maximum the memory throughput by fully utilizing the physical constraints of the memory device, such as read-to-precharge delay (*t*_RTP_), the precharge-to-activate delay (*t*_RP_), and the activate-to-read delay (*t*_RCD_). So it is also not the best choice to design our criticality driven scheduling algorithm in global memory level.

Oppositely, it is necessary and effective to design criticality-aware scheduling algorithm on shared LLC. The LLC scheduling algorithm is trivial in traditional CMP architecture as the memory intensity of CPU applications is moderate. But in GPU architecture, there are numerous SMs to send memory requests to the shard LLC. This leads to severe port confliction on shared LLC. This is an important difference between GPU and traditional CMP architectures. The memory requests from all SMs will aggregate and interact on shared LLC, so adjusting the memory request service sequence in shared LLC level has congenital advantage (LLC port confliction) and acquired advantage (all memory requests aggregate here).

## 4. Critical-Aware LLC Request Scheduling Algorithm (CaLRS)

In this section, we will introduce the proposed critical-aware LLC request scheduling algorithm (CaLRS) in detail. CaLRS improves the SM schedulability by accelerating the critical memory requests. CaLRS exploits the characteristic that different warps may produce different amount of memory requests when executing memory instructions and the constraint that a warp cannot resume to run until all its memory requests have been served to define the criticality of a GPU memory request, that is, the number of memory requests that belong to the same warp and have not been served when it arrives at LLC. CaLRS uses the criticality of a memory request to design the scheduling priority of it in the shared LLC. Specifically, the smaller the criticality is, the higher the priority it has. Therefore, the CaLRS is actually a priority queue management on the shared LLC.

### 4.1. Designing CaLRS

In CaLRS, when a GPU memory request is generated by the coalescing logic, a Critical Field (CF) is appended to mark its criticality. The initial value of CF is set to the number of memory requests that are generated by the memory warp, so all the memory requests that originate from the memory warp have the same original CF value. Suppose that a warp generates *N* memory requests; then each of these *N* memory requests has the same CF value of *N*. Since each GPU memory warp may generate at least 1 memory request and at most 32 memory requests, the value of *N* falls in 1 to 32 (*N* ∈ [1, 32]). So a 6-bit CF is needed to store this information. The CF will decrease when some memory requests are served by GPU's private cache. When the memory requests arrive at shared LLC, the CF will be used to calculate the scheduling priority. As CaLRS is designed below private cache, we have to update the CF value when memory requests pass through the private cache. The update principle is as follows.

*Principle 1*. Each time a memory request hits in the GPU private cache, the CF value of each memory request that originates from the same memory warp decreases by 1 to 0.

If the CF value of a memory request is *N* when it hits in private cache, then the other *N* − 1 memory requests that originate from the warp of the hit one will decrease from *N* to *N* − 1. The reduction of CF will increase their scheduling priority to accelerate their data fetch speed and reduce the memory access latency of the memory warp. Under this update principle, for memory requests generated from the same warp, except the memory request that already hits in private cache, all other memory requests change the CF value in lockstep, so they will always keep the same value.

Formally, the memory request scheduling priorities of CaLRS are designed as (1) requests with lower CF values over requests with higher CF values, (2) among requests with the same CF value, older requests over younger ones. So if a GPU memory request hits in private cache, it will promote the other memory requests' priorities that come from the same memory warp.

### 4.2. Hardware Implementation

**Figure 5:**
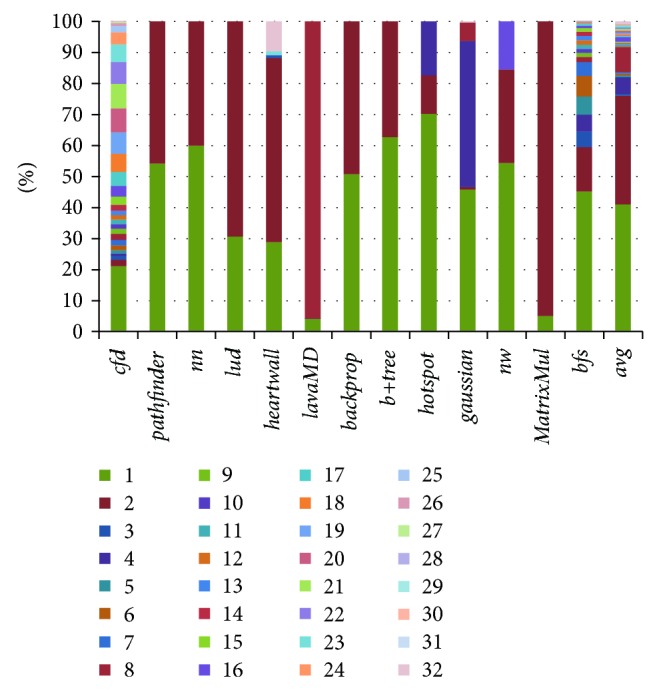
Memory request queue length on shared LLC.

For hardware simplicity, CaLRS uses multiple independent priority subqueues with different priority to buffer the memory requests. As a comparison, we implement a FIFO scheduler in each LLC bank as baseline. According to the value range of CF, a memory request may have at most 32 cases. The statistics of CF value is made in Figure 5. The results show certain regularity of the memory requests. Firstly, in most benchmarks, the memory requests where their CF equals 1, 2, 4, 8, 16, and 32 are overwhelming. On average, their percentages achieve 41.00%, 35.03%, 5.43%, 8.09%, 1.53%, and 0.77%, respectively. Secondly, most benchmarks have only a few types of memory requests according to CF value. Specifically, the number of types is 2 to 5 and their values are just mentioned above. This illustrates the types of memory requests are very concentrated, and their criticality is the power of 2. But* cfd* and* bfs* are two exceptions. On one hand, the sum percentage of the type of memory requests mentioned above is only 29.31% and 67.41%, much less than other benchmarks' 98%. This shows that the other type of memory requests from them cannot be ignored. On the other hand, at these situations, the number of memory request types is more than 5. Specifically, the number of memory request types of* cfd* and* bfs* achieves 30 and 26, respectively. For benchmark* gaussian*, although it has 32 types of memory requests, the three types of memory requests where their CF equal to 1, 4, and 8 occupy over than 98.6%, so we classify it into the regular category.

**Figure 6:**
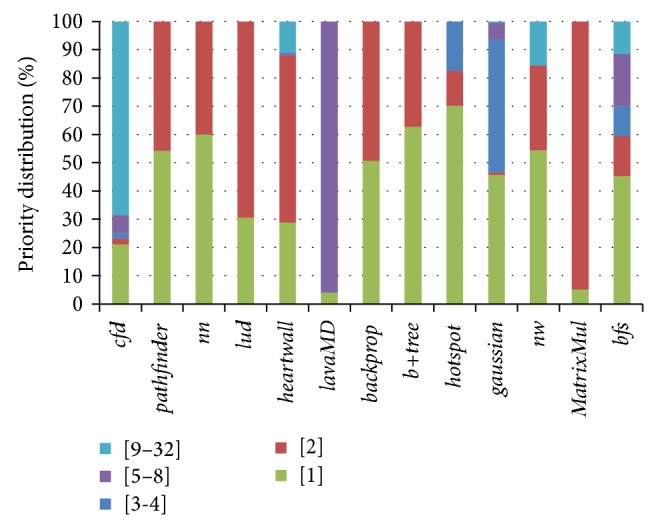
Priority distribution of GPU memory requests.

If we assign an individual scheduling priority for each CF value, then 32 priorities are needed. If an individual subqueue is assigned to each priority, then 32 subqueues are needed to buffer the memory requests of each priority. From the results of Figure 5, we can see that the space utilization of 32 subqueues is very low since most benchmarks do not have so many types of memory requests. Besides, it will be complicated to implement and manage 32 subqueues in hardware. For hardware simplicity, we must decrease the number of subqueues. We achieve this target by decreasing the number of priorities. In view of the fact that the CF values mentioned above are exactly the power of 2, we total up the memory requests with the power of 2. Then there are only 6 categories, namely, [1], [2], [3-4], [5–8], [9–16], and [17–32]. Since the number of memory requests from categories [9–16] and [17–32] is too small to compare with other categories, we combine them into [9–32]. The final result is shown in Figure 6. At this moment, the percentage of each category is 45.2%, 14.2%, 10.5%, 18.6%, and 11.4%.

Therefore, we use 5 priorities to correspond to the 5 categories. As each priority is buffered in an individual subqueue, we implement 5 subqueues (subqueue0~subqueue4). Each priority subqueue is implemented simply as a FIFO queue. These 5 subqueues have priorities from 0 to 4. And the smaller the value is, the higher the priority is. In our baseline architecture, the FIFO queue size is 128, so to keep the same queue size, the sum of the 5 subqueues in CaLRS is also set to 128. In order to avoid starvation, we designed a rotation method, which will be discussed in the next section. Since we employ the rotation method, a skewed subqueue length division method makes no sense and we divide the total size equally among all subqueues. The subqueue lengths and correspondent CF value range are shown in Table 2.

When a GPU memory request arrives at LLC bank, the LLC controller will insert the memory request into the correspondent subqueue according to the request's CF value. If there has no free entry in the correspondent subqueue, then the controller will keep searching the subqueues of lower priority until it finds a free entry and inserts the memory request. If no free entry can be found after searching all lower priority subqueues, then we say the LLC bank is full and we will set a block signal, preventing subsequent memory requests from sending to the bank. This insertion method ensures that no memory request can be inserted beyond its priority. In order to avoid the block signal from joggling, we delay the block signal cancelation. Instead of canceling the block signal once there are free entries (next cycle), we cancel it after all the memory requests in the highest priority subqueue have been served. This cancelation method can coordinate with the rotation method we used to avoid starvation. After all memory requests of this cycle have been inserted, the LLC bank will issue one memory request from the nonempty subqueue of the highest priority in each cycle according to the scheduling priority, keeping at most one-request service at every cycle.

### 4.3. Prevention from Starvation

But starvation may occur using the scheduling method described above. If the new coming memory requests always have high priorities, then the subqueues of high priorities will not be cleared in a long time, which will prevent the memory requests in the subqueues of low priorities from being issued in a long time and finally lead to starvation. Starvation will lead GPU threads to run askew and make thread synchronization costly. To prevent from starving, we designed a simple but effective rotation method to promote the priority of each subqueue circularly. Specifically, when the subqueue of the highest priority, known as subqueue *i* (*i* = 0 in origin), is emptied, we promote the priority by 1 for all other subqueues. Then the priority of subqueue (*i* + 1)%5 becomes 0, subqueue (*i* + 2)%5 becomes 1, and so on. Finally, the priority of subqueue (*i* + 4)%5 is promoted to 3 and the original subqueue *i* is degraded into the lowest priority (priority 4). After such a rotation, the subsequent incoming memory requests with CF equal to 1 will be inserted into subqueue (*i* + 1)%5 and so on. Using this rotation method, the subqueue *i* will be cleared unless all the subsequent memory requests have CF equal to 1. In fact, if all the subsequent incoming memory requests have CF value equal to 1, then any scheduling methods based on CF are meaningless. We did not find any such applications and we are sure that this will not happen in modern GPUs. Otherwise, subqueue of lower priority must become high priority someday and gain prior order to be issued, preventing from starvation.  Algorithm 1 shows the final CaLRS scheduling algorithm.

## 5. Experimental Results

**Figure 7:**
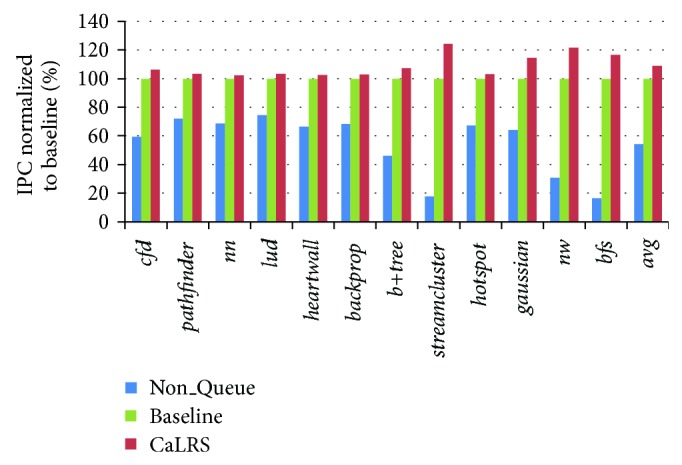
CaLRS performance improvement.

In this section, we will test our CaLRS scheme and analyze the experimental results. The default GPU warp scheduler is GTO.  Figure 7 demonstrates the performance comparison of CaLRS, Non_Queue, and baseline configuration. In the Non_Queue configuration, there is no queue in the shared LLC controller. Instead, it has an 8-size queue in each SM cluster, totaling 128-size buffer. So Non_Queue's buffer size is equal to baseline and CaLRS. We can see that baseline and CaLRS outperform Non_Queue in every benchmark, showing that keeping a request buffer in shared LLC is very beneficial to LLC performance. CaLRS outperforms Non_Queue by 101.4% and baseline by 9.0% in average. Besides, we find that in every benchmark, CaLRS outperforms baseline in different range, which verifies the validity of CaLRS scheme.

**Figure 8:**
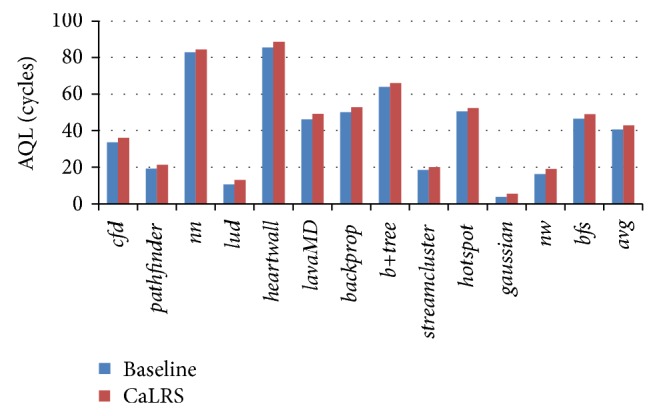
Average queue length comparison of baseline and CaLRS.

Figure 8 shows the average queue length comparison of baseline and CaLRS. We can see that, compared with baseline, the average queue length of all benchmarks is increased in different degree. This is because in baseline, LLC will always accept new memory request unless the queue is full. So the baseline has 100% space utilization of the queue. But in CaLRS scheme, in the situation where subqueues with lower priorities are full but lower ones still have free entries, memory requests with low priority will be rejected and block LLC. So some entries in lower priority subqueues will not be utilized efficiently. Overall, a memory request can enter LLC bank queue a bit earlier and has shorter average queue latency (as large as the queue length) in baseline. In average, CaLRS's average queue latency increases by 2.29 clock cycles (about 5.65% of baseline), which is the time overhead of CaLRS.

**Figure 9:**
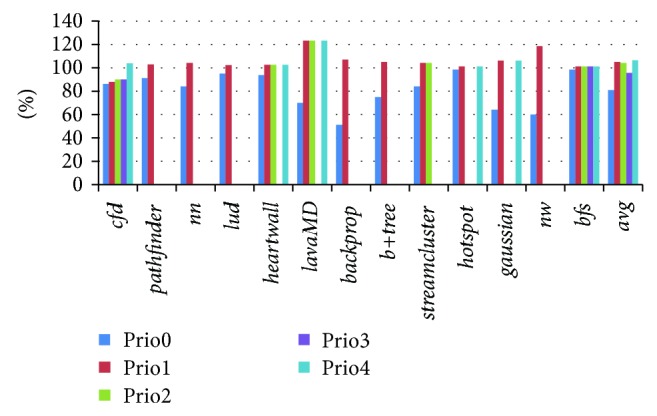
The average queue latency changement for different classes of memory requests that have the same priority.

The idea behind CaLRS is to reduce the queue latency of high priority memory requests at the expense of low priority memory requests.  Figure 9 illustrates the average queue latency changement for each class of memory requests that have the same priority. Most benchmarks do not have all classes of memory requests, which is dependent on the intrinsic memory accessing character of benchmarks and our classification method. In CaLRS, the queue latency for memory requests with lowest CF values becomes smaller as they are prioritized. On the other hand, the memory requests with the highest CF values will definitely be delayed and their queue latency becomes larger. For other memory requests, the situation is uncertain, depending on the memory request distribution of the 5 kinds. The results show that, in CaLRS, the average queue latency of each class with priority from 0 to 4 becomes 80.83%, 105.07%, 104.18%, 95.60%, and 106.31% compared with baseline, respectively. The results reflect the idea of CaLRS visually.

**Figure 10:**
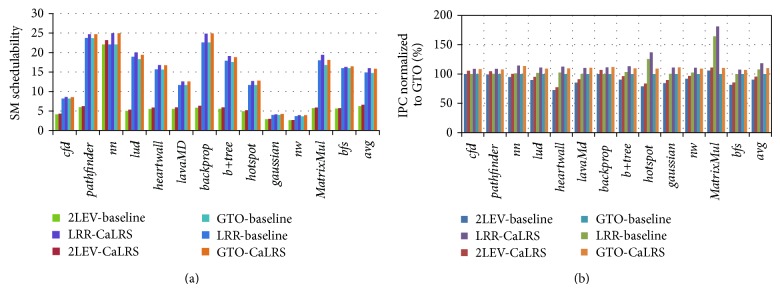
CaLRS performance under various warp scheduling algorithms: (a) SM schedulability, (b) overall IPC.

Figure 10 shows how CaLRS improves the SM schedulability and overall performance under different warp scheduling algorithms, in which “2LEV” stands for two-level scheduler [8], “LRR” stands for loose round robin scheduler, and “GTO” stands for greedy then oldest scheduler. The results are normalized to “GTO-”baseline. From Figure 10(a), we find that different warp scheduling algorithms provide various SM schedulability, in which “2LEV” falls far behind than the other two. Secondly, by comparing the results of baseline and CaLRS, we can see that the SM schedulability is obviously improved in all of the three warp scheduling algorithms. Specifically, “2LEV,” “LRR,” and “GTO” improved SM schedulability by 15.21%, 17.53%, and 17.69%, respectively. From Figure 10(b), we can see that the “2LEV” scheduler has the lowest performance than the other two, which is compatible with the result of SM schedulability. By comparing the results of baseline and CaLRS, we can see that CaLRS outperforms baseline in certain degrees in each warp scheduling algorithm. Specifically, CaLRS improves by 5.88%, 9.79%, and 10.63% for “2LEV,” “LRR,” and “GTO,” respectively. The benchmark* MatrixMul* in “LRR” scheduler has the most performance improvement, reaching 80.95%. By comparing these three warp scheduling algorithms, we see that the GPU performance is positive proportional to the SM schedulability.

**Figure 11:**
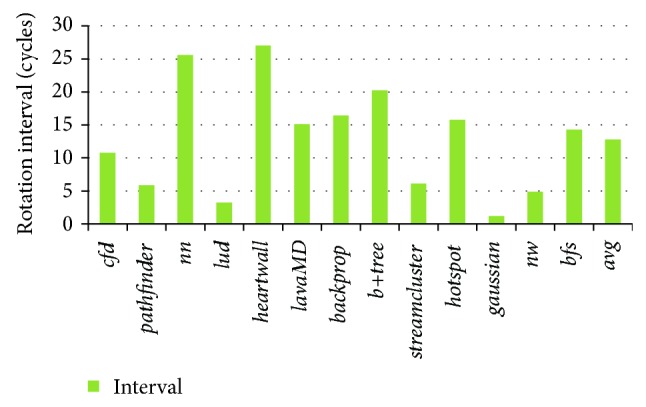
Average rotation interval in CaLRS.

According to the rule, rotation can only happen when the highest priority subqueue becomes empty.  Figure 11 tests the average rotation interval in CaLRS. We can see that the rotation executes once in every 12.8 cycles in average. The benchmarks* heartwall* and* nn* have the longest rotation interval as they have intensive memory access rate and high percentage of Prio0 memory requests from Figures 3 and 6, respectively. The percentage of high priority memory requests contributes to long interval as more new incoming memory requests have high priority and insert into the highest subqueue, which prevent the subqueue from clearing and thus rotating. When the rotation finishes a round, any memory requests buffered in the subqueues must have been served. So the maximum queue latency must be smaller than rotation intervals multiplied by five.

**Figure 12:**
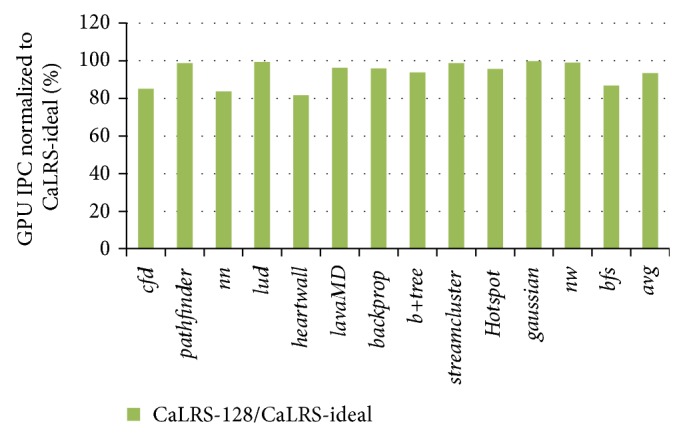
Performance comparison of CaLRS-128 and CaLRS-ideal.

We have also compared the performance of hardware implemented CaLRS and ideal CaLRS to test the performance gap between them. The ideal CaLRS have 32 subqueues and each subqueue has infinite large size. We call the CaLRS with 5 subqueues and 128-size as CaLRS-128 and the ideal one as CaLRS-ideal.  Figure 12 is the performance comparison of CaLRS-128 and CaLRS-ideal. We can see from the figure that CaLRS-128 achieves up to 90% of CaLRS-ideal except benchmarks* cfd*,* nn*,* heartwall*, and* bfs*. And the average value is 93.38%, which is pretty good already. The CaLRS-ideal will outperform CaLRS-128 in the following aspects. The first is that the infinite large subqueue can prevent LLC from blocking because of buffer full. The second is that the larger number of subqueues enables CaLRS to recognize the criticality nuances of memory requests. The rotation scheme will disturb the normal order as it will promote lower ones regardless of CF value. We find that benchmarks* nn *and* heartwall* go into the first case and* cfd* and* bfs* go into the second case. Benchmarks* nn *and* heartwall* are memory very intensive benchmark in which the subqueues may become full occasionally, thus preventing following memory requests from inserting to the queues. In benchmarks* cfd* and* bfs*, there are more than 20 classes of memory requests according to the CF value, so the rotation will influence them severely. Overall, although CaLRS-ideal is better than CaLRS-128, the gap is not large. On the other hand, CaLRS-ideal is infeasible in hardware, neither the infinite large buffer size nor managing as many 11 as 32 subqueues in one single cycle.

## 6. Related Work

### 6.1. Memory Scheduling Techniques

In the traditional multicore areas, there is a lot of research work for memory scheduling techniques. Kim et al. [9] and Ebrahimi et al. [10] proposed a variety of memory request priority methods to improve the fairness and throughput for multiple single-thread programs and paralleled applications. But in the GPU fields, there are only a few works since this is a new research area. Yuan et al. [11] proposed a method that provides an arbitration mechanism at the interconnection network to store these memory access requests losing the row buffer locality. They showed that the mechanism enables in-order DRAM memory scheduler's performance to be approximate to FR-FCFS [12, 13]. Lakshminarayana et al. [14] developed a DRAM scheduling policy that chooses a method between Shortest Job First (SJF) and FR-FCFS. Ausavarungnirun et al. [15] proposed a phased memory scheduler for CPU-GPU architecture. Their main objective is to enhance row-buffer locality in heterogeneous architectures. Jog et al. [16] focused on multiple applications running simultaneously on the GPU and proposed a method by adding round robin manner on the basis of FR-FCFS to improve fairness and performance.

### 6.2. Warp Scheduling Techniques

Fung el al. [3] proposed dynamic warp reformation scheme in which the threads that have similar execution time in different warps are extracted and formatted into a new warp, thus overcoming the threads synchronization cost and latency between original warps. Gebhart et al. [17] proposed a two-level warp scheduling technique that focuses on reducing the energy consumption in GPUs. Chen et al. [18] proposed a novel warp scheduling algorithm that flexibly uses the time slice round-robin feature to utilize GPU parallelism. Jog et al. [19] and Kayiran et al. [20] proposed CTA-aware warp scheduling algorithms to reduce cache and memory contention or improve thread-level parallelism. Rogers et al. [4] analyzed how hardware scheduler influences the management to GPU cache and proposed a cache sensitive warp scheduling policy. A local detector is used to collect the locality due to cache capacity contention. Jog et al. [21] proposed a prefetching-aware warp scheduling policy to separate in time the scheduling of consecutive warps such that they are not executed back-to-back. Kuo et al. [22] proposed a thread scheduling algorithm according to cache capacity contention.

### 6.3. GPU Cache Optimization Techniques

Memory access coalescing of GPGPU also has a great impact on memory access performance and cache data blocks' reuse degree. Wang [23] proposed a cache management strategy that prioritizes the data block requests with either low access density or low divergence. Mu et al. [24] proposed a cache management strategy that prioritizes the cache block with more valid addresses and a memory scheduling strategy that first responses to the warp request which has less memory accesses. Sankaranarayanan et al. [25] introduce a smaller tinyCache for every processing unit over L1 to reduce the energy consumption of L1 and scratchpad memory. Since it does not need memory access coalescing and many ports to connect to multiple processing units, it does not need multiport or multibank and its access cost is far less than that of L1.

## 7. Conclusion

In this paper, we observed that there are a large number of GPU memory requests queuing in the shared LLC banks. The warp-agnostic LLC scheduling situation leads to suboptimal performance. We find that the SM schedulability can be improved by adjusting the memory request service sequence properly. So we designed a memory request scheduling algorithm on shared LLC based on the criticality of memory request. The criticality of GPU memory request is defined as the number of memory requests to be served that originate from the same warp when arrival at LLC bank. The proposed CaLRS promotes high critical memory requests at the expense of delaying others. Experiments show that the proposed CaLRS can improve the SM schedulability on several present warp scheduling algorithms and then improve the GPU performance. We believe that the memory request control on shared LLC will become even important when the core number increases in GPUs and more irregular applications are ported onto GPUs.

## Figures and Tables

**Algorithm 1:**
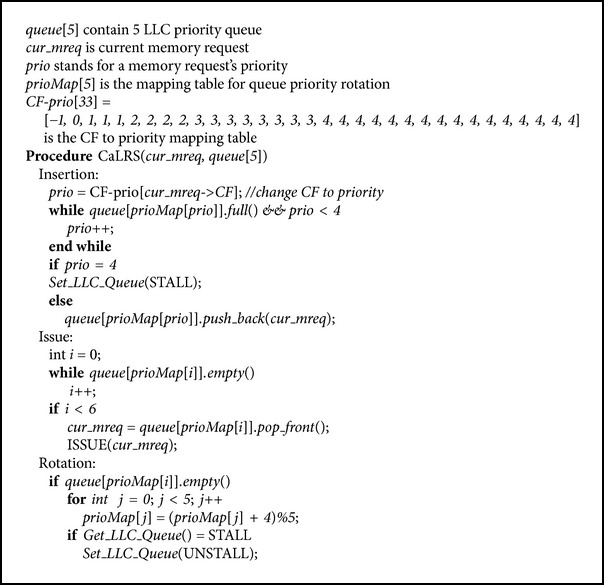
CaLRS scheduling algorithm.

**Table 1:** Other configurations of baseline GPU.

# of SMs	30 (15 clusters of 2)
SM configuration	1400 Mhz, Reg #: 32K, SIMD Width: 16, warp: 32 threads, and max threads per SM: 1536
Branching handling	PDOM based method [3]
Warp scheduling	Greedy-then-oldest (GTO) [4]
Private L1 caches	16 KB $L1D, 8 KB $Const, 12 KB $Texture, and 2 KB $L1I
Scratchpad memory	48 KB
Interconnect	Butterfly, 1400 Mhz, 32 B width
# of LLC banks	6 (= #of memory partitions)
LLC bank controller	First-in-first-out (FIFO)
LLC unified cache	768 KB, 128 B line, and 8-way
Min. LLC latency	120 cycles (compute core clock)
Memory controller	Out-of-order (FR-FCFS), max request queue length: 32
GDDR5 timing (from Hynix H5GQ1H24AFR)	*t*_CCD_ = 12, *t*_CL_ = 12, *t*_RP_ = 12, *t*_RC_ = 40, *t*_RAS_ = 28, *t*_RCD_ = 12, *t*_RRD_ = 6, and *t*_CDLR_ = 5, *t*_WR_ = 12
Min. DRAM latency	460 cycles (compute core clock)

**Table 2:** Subqueues, CF, and priority correspondence.

Subqueue name	Length	CF value range	Priority
Subqueue0	25	1	0
Subqueue1	25	2	1
Subqueue2	25	3~4	2
Subqueue3	25	5~8	3
Subqueue4	28	9~32	4
